# Respiratory chain signalling is essential for adaptive remodelling following cardiac ischaemia

**DOI:** 10.1111/jcmm.15043

**Published:** 2020-02-10

**Authors:** Marten Szibor, Rolf Schreckenberg, Zemfira Gizatullina, Eric Dufour, Marion Wiesnet, Praveen K. Dhandapani, Grazyna Debska‐Vielhaber, Juliana Heidler, Ilka Wittig, Tuula A. Nyman, Ulrich Gärtner, Andrew R. Hall, Victoria Pell, Carlo Viscomi, Thomas Krieg, Michael P. Murphy, Thomas Braun, Frank N. Gellerich, Klaus‐Dieter Schlüter, Howard T. Jacobs

**Affiliations:** ^1^ Faculty of Medicine and Health Technology Tampere University Tampere Finland; ^2^ Institute of Biotechnology University of Helsinki Helsinki Finland; ^3^ Department of Cardiothoracic Surgery Jena University Hospital Jena Germany; ^4^ Department of Physiology Justus‐Liebig University Giessen Giessen Germany; ^5^ Department of Neurology Otto‐von‐Guericke‐University Magdeburg Germany; ^6^ Department Cardiac Development and Remodelling Max Planck Institute for Heart and Lung Research Bad Nauheim Germany; ^7^ Functional Proteomics Faculty of Medicine Goethe University Frankfurt am Main Germany; ^8^ Department of Immunology Institute of Clinical Medicine Oslo University Hospital University of Oslo Oslo Norway; ^9^ Institute of Anatomy and Cell Biology Justus‐Liebig‐University Giessen Giessen Germany; ^10^ Medical Research Council Mitochondrial Biology Unit University of Cambridge Cambridge UK; ^11^ Department of Medicine University of Cambridge Cambridge UK; ^12^ Department of Biomedical Sciences University of Padova Padova Italy

**Keywords:** adaptive cardiac remodelling, alternative oxidase, cardiac ischaemia‐reperfusion, electron transport chain, mouse, reactive oxygen species

## Abstract

Cardiac ischaemia‐reperfusion (I/R) injury has been attributed to stress signals arising from an impaired mitochondrial electron transport chain (ETC), which include redox imbalance, metabolic stalling and excessive production of reactive oxygen species (ROS). The alternative oxidase (AOX) is a respiratory enzyme, absent in mammals, that accepts electrons from a reduced quinone pool to reduce oxygen to water, thereby restoring electron flux when impaired and, in the process, blunting ROS production. Hence, AOX represents a natural rescue mechanism from respiratory stress. This study aimed to determine how respiratory restoration through xenotopically expressed AOX affects the re‐perfused post‐ischaemic mouse heart. As expected, AOX supports ETC function and attenuates the ROS load in post‐anoxic heart mitochondria. However, post‐ischaemic cardiac remodelling over 3 and 9 weeks was not improved. AOX blunted transcript levels of factors known to be up‐regulated upon I/R such as the *atrial natriuretic peptide* (*Anp*) whilst expression of pro‐fibrotic and pro‐apoptotic transcripts were increased. Ex vivo analysis revealed contractile failure at nine but not 3 weeks after ischaemia whilst label‐free quantitative proteomics identified an increase in proteins promoting adverse extracellular matrix remodelling. Together, this indicates an essential role for ETC‐derived signals during cardiac adaptive remodelling and identified ROS as a possible effector.

## 1 INTRODUCTION

The high susceptibility of cardiomyocytes to oxygen deprivation has been originally attributed to their almost exclusive dependence on oxidative metabolism for ATP production.^1^ Indeed, approximately 30% of the cardiomyocyte's intracellular volume is occupied by mitochondria which harbour the electron transport chain (ETC) in their inner membrane. The ETC is composed of four oxidoreductase complexes (cI‐cIV) that facilitate substrate oxidation and electron transfer with oxygen being the final acceptor. Importantly, electron transfer is coupled to the generation of a proton‐electrochemical potential gradient across the inner mitochondrial membrane, which itself is the driving force for ATP production, a process described as oxidative phosphorylation (OXPHOS). Although the ETC thus couples ATP production to oxygen consumption, pioneering work in isolated rabbit heart mitochondria^2^ as well as in intact rabbit hearts^3^ revealed that cardiac ATP depletion alone is insufficient to account for all post‐ischaemic tissue damage.^2^ Instead, electron flow through the ETC upon reperfusion was described as a source of potentially detrimental reactive oxygen species (ROS), proposed to trigger ischaemia‐reperfusion (I/R) injuries.^2^ Conversely, the therapeutic use of untargeted antioxidants such as vitamin C for post‐ischaemic cardioprotection has given contradictory results,^4^, ^5^, ^6^, ^7^, ^8^, ^9^ and itself produced detrimental side effects when therapeutically used.^10^ These seemingly contradictory results were thought to be due to biphasic effects of ROS with high ROS concentrations leading to damage and lower concentrations eliciting adaptive responses.^11^, ^12^ The term 'mitohormesis' was proposed^13^ to describe this phenomenon. Indeed, a recent study demonstrated that increased ROS levels specifically in the mitochondrial compartment have a cardioprotective effect upon I/R.^14^ Of course, it could equally be possible that untargeted antioxidants cannot reach sufficient levels in the mitochondrial compartment to be effective, and therefore fail to detoxify mitochondrial ROS at the site of its production. Furthermore, ROS may generate positive or negative effects in a time‐dependent manner.

To test this assumption, a number of mitochondrial‐targeted antioxidants have been designed. MitoQ, one prominent example, consists of ubiquinol (reduced quinone) targeted specifically to mitochondria by a covalently fused lipophilic triphenyl phosphonium (TPP) cation.^15^ Importantly, in rats that underwent cardiac I/R, MitoQ, but not the untargeted antioxidant or TPP alone, significantly decreased contractile dysfunction, cell death and mitochondrial damage.^15^ Positive effects of MitoQ were also seen in other studies addressing post‐transplantation injury^16^ and pressure overload‐induced heart failure.^17^ Following a similar rationale, Szeto‐Schiller (SS) peptides^18^ were used, which accumulate in mitochondria in a membrane potential‐independent manner. The use of SS peptides caused phenotypic improvements in a model of angiotensin II‐induced mitochondrial ROS production and myocardial contractile failure^19^ as well as upon transverse‐aortic constriction.^20^ Both, MitoQ and SS peptides appeared biologically safe and beneficial when long‐term administered^21^, ^22^ arguing that intramitochondrial ROS are rather detrimental than physiologically relevant. Finally, using high‐throughput chemical screening, small molecules that suppress superoxide and/or hydrogen peroxide production at the I_Q_ site of cI were identified, S1QELs,^23^ that act through a mechanism that does not affect regular OXPHOS. Most importantly, ROS generated at I_Q_ was discussed to be instrumental for cellular stress signalling. Indeed, application of S1QELs protected the perfused mouse heart from I/R injuries.^23^ A unifying mechanism by which the different mitochondrially targeted antioxidants may fulfil their beneficial effects was recently put forward.^24^ According to this, succinate, an intermediate metabolite of the tricarboxylic acid cycle, accumulates during cardiac ischaemia.^25^ Reperfusion evokes rapid succinate oxidation through reverse electron transport (RET) from ubiquinol to cI concomitant with a burst of mitochondrial ROS. Scavenging RET‐induced ROS may therefore be the mechanism by which cellular and organ damage is prevented although other sources of ROS, that are intrinsically linked, may play a signalling role as well.^26^

Plants and many other organisms, but not insects or vertebrates, harbour a by‐pass mechanism to protect from ETC‐mediated respiratory stress conditions, the alternative oxidase (AOX).^27^ AOX is a non‐protonmotive single di‐iron carboxylate redox transfer protein.^28^ Where expressed, AOX oxidizes ubiquinol and by‐passes cIII and cIV, directly transferring electrons to oxygen. In previous studies, we were able to show that a xenotopically expressed AOX from the tunicate *Ciona intestinalis* (hereafter called AOX) is catalytically active under various experimental conditions. In human cells, AOX conferred resistance against the ETC inhibitor cyanide^29^ and corrected genetic defects which caused ETC dysfunction.^30^ In fruit flies, AOX complemented ETC defects in vivo and restored viability.^31^ Global expression in the mouse protected against cyanide toxicity^32^, ^33^ decreased lethality from endotoxemia^34^ and alleviated cigarette smoke‐induced lung remodelling and cell death.^35^ AOX was demonstrated to effectively decrease RET‐induced ROS^36^, ^37^ driven by succinate. Most importantly, however, expression of AOX prevented the development of a lethal cardiomyopathy in a cIII mouse mutant strain^38^ indicating sufficient expression for full respiratory restoration in the stressed heart. Interestingly, in a mouse model of inflammatory cardiomyopathy,^39^ AOX expression in cardiomyocytes conferred detrimental effects.^40^ This suggests a role for the mitochondrial respiratory chain in both, energy homeostasis and stress signalling.

Here, we tested how maintaining electron flow impacts on remodelling in the post‐ischaemic mouse heart. We show that AOX expression supports ETC function and decreases mitochondrial ROS levels, yet impairs adaptive cardiac remodelling. This indicates an essential role for ETC‐derived stress signals in post‐ischaemic cardiac adaptation.

## 2 MATERIALS AND METHODS

### 2.1 Animal models

Mice with ubiquitous expression of *Ciona intestinalis* AOX^33^ were maintained on a C57BL/6J background in temperature‐ and humidity‐controlled facilities at 12 hours of light‐to‐dark cycles with access to water and food ad libitum. Open‐chest in situ I/R procedures were conducted at the University of Cambridge, Cambridge, UK upon approval by Home Office license 70/8238. In vivo I/R procedures were conducted at the Max Planck Institute for Heart and Lung Research in Bad Nauheim, Germany upon approval by the Regierungspräsidium Darmstadt (V54‐19c20/15‐B2/1014).

### 2.2 Open‐chest in situ I/R mouse model and estimation of infarct size and zone at risk

Details of the procedure have been published elsewhere.^14^, ^41^ Briefly, WT and AOX transgenic littermates were anesthetized and ventilated with ambient air supplemented with oxygen (peak inspiratory pressure of 10 mbar and positive end‐expiratory pressure of 3 mbar) throughout the experiment. The ventilation settings were adjusted to a frequency of 110 hubs per minutes and a tidal volume of 200‐250 µL. Left anterolateral thoracotomy was performed to visualize the mouse heart and lay open the left anterior descending artery (LAD). The LAD was then surrounded by a 7‐0 nylon suture to form a snare. After a 15 minutes period of stabilization, the I/R procedure was started with 30 minutes of ischaemia followed by 2 hour reperfusion. After the I/R procedure, the LAD was re‐occluded and Evans blue was injected retrogradely through the aortic root. Evans blue staining was used to demarcate the zone of ischaemia hereafter referred to as the region at risk zone. After staining, hearts were excised, perfused with 0.9% saline solution, frozen and transversely cut into 1 mm slices. The slices were incubated in 1% triphenyltetrazolium chloride (TTC) in sodium phosphate buffer (pH 7.4) and incubated at 38°C for 20 minutes. TTC stains the viable (non‐infarcted myocardium) brick‐red based on catalytic dehydrogenase activity. Finally, the slices were immersed in 10% formalin and areas of infarct and risk zone determined slice by slice using planimetry.

### 2.3 In vivo I/R mouse model

Male WT and AOX littermates received 0.1 mg/kg bodyweight buprenorphine *s.c.* prior to anaesthesia to minimize postoperative pain. For anaesthesia induction, mice were exposed to 4.5 Vol% isoflurane in ambient air for approximately 3 minutes in an air‐tight box. Anaesthesia was maintained after intra‐tracheal intubation and ventilation using 1.5 Vol% isoflurane using the rodent MiniVent ventilator (Harvard Apparatus, HSE) adjusted to 225 hubs per minutes with a tidal volume of 250 µL. During the procedure, animals were kept in a supine position on a heat‐controlled plate at 37°C. A left anterolateral thoracotomy was performed between the second and third rib to visualize the mouse heart and lay open the LAD. The LAD was ligated in a proximal position using a 7‐0 prolene suture. Pale discoloration of ventricular tissue demarcated the region of ischaemia. After ligation, animals received 5 IU of Heparin‐Natrium s.c. and the open wound was covered using cheese cloth soaked with 0.9% NaCl solution. After 45 minutes of ischaemia, the ligation was opened and cardiac reperfusion confirmed by visual control. The wound was closed using absorbable, synthetic 5‐0 vicryl (polyglactin 910) sutures. Weaning from ventilation took several minutes and was ended when spontaneous respiration became evident.

### 2.4 Langendorff perfusion experiments

Hearts were isolated from mice, and procedures to measure cardiac functions were performed as previously described.^42^ Briefly, after deep anaesthesia with isoflurane (5%), hearts were excised from the chest cavity, transferred rapidly to ice‐cold saline and immediately mounted on the cannula of Langendorff perfusion system. Heart perfusion and all next steps were done at 37°C. Perfusion of hearts was performed with perfusate (in mmol/L: NaCl 118, KCl 4.7, KH_2_PO_4_ 1.2, KCl 2.5, MgSO_4_ 0.8, NaHCO_3_ 25, glucose 5, C_3_H_3_NaO_3_ 1.9, CaCl_2_ 2.5). Perfusate flow was adjusted to a perfusion pressure of 70 mm Hg. To determine left ventricular pressure, a balloon was inserted into the left ventricle and inflated at 10 mm Hg. Ischaemia was initiated by flow stop.

### 2.5 Respirometry and ROS production using isolated mitochondria

Mitochondria were isolated from cardiac tissue as described previously.^43^ Mitochondrial oxygen consumption and ROS production were measured using an Oroboros O2k oxygraph (Oroboros Instruments) at 37°C. Standard experimental set‐up was 200 µg of mitochondrial protein per mL of mitochondrial respiration buffer (120 mmol/L KCl, 1 mmol/L EGTA, 10 mmol/L HEPES, 5 mmol/L KH_2_PO_4_, pH 7.2; Sigma, P5405, E3889, H3375, P5655) along with 30 μL of 1 mg/mL superoxide dismutase (Sigma, S5395), 30 µL of 1 mg/mL horseradish peroxidase (Sigma, 77332), 30 µL of 10 mg/mL fatty acid‐free‐BSA (Sigma, A6003) and 5 µL of Amplex Red Reagent (ThermoFisher, A12222) for hydrogen peroxide detection. Mitochondrial respiration was initiated by the addition of 10 mmol/L succinate (Sigma, S3674). To confirm AOX activity, 1 mmol/L cyanide (KCN, Sigma, 60178) was added to both WT and AOX mitochondria, prior to AOX inhibition by 100 μM *n*‐propyl gallate (Sigma, P3130). To assess hydrogen peroxide production as the consequence of RET, mitochondrial respiration was stimulated by the addition of succinate and respiration allowed to diminish all oxygen present within the chamber. Once anoxic, mitochondria were left for 20 or 30 minutes to simulate ischaemia, after which oxygen was allowed back into the chamber and oxygen consumption and ROS production measured. To assess sole AOX activity, cyanide was added at the start of the experiment.

### 2.6 Western blot analysis

Western blots were performed essentially as described elsewhere.^44^ Rabbit serum raised against two AOX peptides (anti‐AOX, 1:20 000, 21st Century Biochemicals)^30^ and mouse monoclonal antibody against the voltage‐dependent anion channel (anti‐VDAC1, 1:1000, Abcam ab14734) were used and protein bands were visualized using a LI‐COR Odyssey flatbed scanner with anti‐mouse and anti‐rabbit secondary antibodies conjugated to IRDye 680RD and IRDye 800CW, respectively.

### 2.7 Respirometry using skinned heart fibres and heart tissue homogenate

Mitochondrial respiratory activity upon in vivo I/R was measured using skinned fibre isolation (3‐weeks I/R) as described in detail elsewhere^45^, ^46^ or after homogenization of heart tissue using a POLYTRON PT 1200 E Manual Disperser (Ecoline; 9‐weeks I/R). For skinned fibre isolation, left‐ventricular heart tissue was dissected on ice in a plastic cell‐culture dish using extra sharp forceps to extract thin muscle fibres. These fibres were permeabilized for 30 minutes using saponin (50 µg/mL) as previously described.^45^, ^46^ Upon washing, fibres were dried on Whatman paper and weighted before transfer to the oxygraph chamber. For heart tissue homogenization, left‐ventricular heart tissue was weighted and then minced on ice using scissors and a POLYTRON homogenizer before transfer to the oxygraph chamber. Using either technique, mitochondria were found to be accessible for substrates and inhibitors, and exhibited coupled respiration albeit at different activity levels per mg of the respective sample. Oxygen consumption was measured using Oroboros O2k oxygraphs (Oroboros Instruments, Innsbruck, Austria)^47^ at 30°C calculated from the time derivative of the oxygen concentration using DatLab 7 software (Oroboros Instruments, Innsbruck, Austria). 1‐2 mg of fibre/tissue homogenate was added per chamber to respiration buffer containing 120 mmol/L D‐mannitol (Sigma‐Aldrich, M4125), 20 mmol/L MOPS (Sigma, M1254), 5 mmol/L KH_2_PO_4_ (Sigma, P5655), 60 mmol/L KCl, 5 mmol/L MgCl_2_, pH7.4. Substrates used for cI‐driven respiration (PGM plus ADP): 10 mmol/L pyruvate (P, Sigma, P5280), 10 mmol/L glutamate (G, Aldrich, 49621), 2 mmol/L malate (M, Sigma, M1000) and adenosine 5′‐diphosphate (ADP, Sigma, A2754). Substrates used for cIV measurements: 2 mmol/L (+)‐sodium L‐ascorbate (Asc, Sigma, A7631) and 0.5 mmol/L *N*,*N*,*N*′,*N*′‐tetramethyl‐*p*‐phenylenediamine (TMPD, Sigma, T7394). Respiratory inhibitors used for control: 1.5 µmol/L rotenone (Sigma‐Aldrich, R8875), 5 mmol/L sodium azide (Sigma‐Aldrich, S2002), 1 mmol/L cyanide (Sigma, 60178) and 100 µmol/L *n*‐propyl gallate (Sigma, P3130).

### 2.8 RNA extraction, reverse transcription (RT) and qPCR

Total RNA was extracted from left ventricles by RNeasy Micro Kit (Qiagen). The extracted RNA was reverse‐transcribed (RT) to cDNA using iScript cDNA Synthesis Kit (Bio‐Rad). qPCR was done using iTaq SYBR Green Supermix with ROX (Bio‐Rad) and Mx3000P qPCR System (Agilent Technologies). All measurements were carried out in duplicate. Melting curves confirmed desired products formation. The calculation of ΔCt values was done by subtracting the Ct values of the target gene from the endogenous control [ΔCt = Ct (endogenous control) − Ct (target)]. Transcript expression levels as fold change were calculated from 2^−ΔΔCt^. *B2m* (*β*_2_ *microglobulin*) served as a reference housekeeping gene.

**Table:** 

Gene	Forward primer	Reverse primer
*B2m*	GCTATCCAGAAAACCCCTCAA	CATGTCTCGATCCCAGTAGACGGT
*Anp*	GGCTCCTCCCTCGTCTTGG	GCTTCCTCAGTCTGCTCAC
*Col1a1*	TTCTCCTGGAAAGATGGTGC	GGACCAGCATCACCTTTAACA
*Col3a1*	CTTTCCCGGTGGGCGTGGTC	TGAGCACCAGGTGGCCCCTT
*Sod2*	GACTATGGCGCGCTGGAGCC	TCCCTTGGCCAGAGCCTCGT
*Ucp2*	GGCCTCTGGAAAGGGACTTC	GACCACATCAACAGGGGAGG
*Bax*	TACAGGGTTTCATCCAGG	ATTGCTATCCAGCCTATCTC
*Bcl2*	TCGCAGAGATGTCCAGTC	CCCACCGAACTCAAAGAAG

### 2.9 Transmission electron microscopy

A small piece of tissue from the heart apex was kept in storage buffer containing 1.5% glutaraldehyde, 1.5% formaldehyde, 0.15 mol/L HEPES/KOH (pH 7.4) at 4°C until embedding and sectioning. For epoxy resin embedding, samples were fixed in 1% osmium tetroxide, stained *en bloc* in half‐saturated uranyl acetate, dehydrated in an ascending ethanol series and embedded in Agar 100 (Agar Scientific). Ultra‐thin sections were cut using an ultramicrotome (Reichert Ultracut E, Leica) and examined in a transmission electron microscope (Zeiss EM 902). Digital images were captured with a slow‐scan 2K CCD camera (TRS, Tröndle, Moorenweis, Germany).

### 2.10 Nano LC‐MS/MS and data analysis

Mouse heart samples were homogenized using a FastPrep‐24 5G High Speed Homogenizer (MP Biomedicals). Tissue pieces were transferred to 2 mL tubes with Lysing matrix D, and 750 µL 8 mol/L urea (Sigma, U5378) in 50 mmol/L NH_4_HCO_3_ (Sigma, A6141) and protease inhibitor mix (cOmplete ULTRA Tablets, 05892970001, Roche Applied Science) were added. Homogenization was done with five cycles of 30 seconds 6 m/s pulses and had 5 minutes break on ice in between the cycles. After homogenization, the protein concentration was measured using NanoDrop, and a volume corresponding to 200 µg protein was used for further analysis. Proteins were reduced with dithiothreitol (DTT, Sigma, D9779), alkylated with iodoacetamide (Sigma, I6125) and in‐solution digested with LysC (2 hours at room temperature, Wako, 125‐05061) and trypsin (overnight at room temperature, Promega, V5111).

The resulting peptides were desalted and concentrated before mass spectrometry using µC18 tip (Pierce C18 Tips). The peptides were eluted with 0.1% trifluoroacetic acid (TFA, Merck, 0.08260.0101) in 60% acetonitrile (ACN, Merck, 1.00029.2500), dried and solubilized in 7 μL 0.1% formic acid (FA, Merck, 1.59013.2500) for mass spectrometry analysis. Each peptide mixture was analysed on an Easy nLC1000 nano‐LC system connected to a quadrupole Orbitrap mass spectrometer (QExactive, ThermoElectron, Bremen, Germany) equipped with a nano‐electrospray ion source (EasySpray, Thermo). For the liquid chromatography separation of the peptides, an EasySpray column capillary of 25 cm bed length was employed. The flow rate was 300 nL/min, and the peptides were eluted with a 2%‐30% gradient of solvent B in 240 minutes. Solvent A was aqueous 0.1% FA and solvent B 0.1% FA in acetonitrile. The data‐dependent acquisition automatically switched between MS and MS/MS mode. Survey full scan MS spectra were acquired from a mass‐to‐charge ratio (*m*/*z*) of 400 to 1200 with the resolution *R* = 70 000 at *m*/*z* 200 after accumulation to a target of 3 000 000 ions in the quadruple. For MS/MS, the ten most abundant multiple‐charged ions were selected for fragmentation on the high‐energy collision dissociation cell at a target value of 100 000 charges or maximum acquisition time of 100 ms The MS/MS scans were collected at a resolution of 17 500. Target ions already selected for MS/MS were dynamically excluded for 30 seconds.

The resulting MS raw files were submitted to the MaxQuant software (version 1.5.7.4)^48^ for protein identification and quantitation using the Andromeda search engine. MaxQuant search was done against the UniProt mouse database (March 2017). Carbamidomethyl (C) was set as a fixed modification and protein N‐acetylation and methionine oxidation were set as variable modifications. First search peptide tolerance of 20 ppm and main search error 4.5 ppm were used. Trypsin without proline restriction enzyme option was used, with two allowed miscleavages. The minimal unique + razor peptides number was set to 1 and the allowed false discovery rate (FDR) was 0.01 (1%) for peptide and protein identification. Label‐free quantification (LFQ) was done with default settings in MaxQuant. The mass spectrometry proteomics data have been deposited to the ProteomeXchange^49^ Consortium via the PRIDE^50^ partner repository with the dataset identifier: PXD014061 and the project name ‘Expression of *Ciona intestinalis* AOX in a mouse model of cardiac ischemia‐reperfusion’.

### 2.11 Statistical analyses

Statistical analyses, except for proteome, were performed using GraphPad Prism (GraphPad Software, version 7 for Mac OS X). 1way or 2way ANOVA and post hoc analyses were used as indicated for comparisons of at least n = 3 independent experiments, and a *P* value <.05 was considered being statistically significant. All data are shown as mean. Error bars represent standard error of the mean (SEM). Proteome analysis was done using Perseus software (version 1.6.1.3).^51^ LFQ values were log2 transformed and filtered to include only proteins identified and quantified in at least three out of five replicates in at least one experimental group, and missing values were imputed with values representing background from a normal distribution with default settings in Perseus. To find statistically significant differences between the sample groups Student's *t* test was done with a *P* value of <.05 being considered statistically significant.

## 3 RESULTS

### 3.1 AOX does not decrease acute I/R injuries

**Figure 1:**
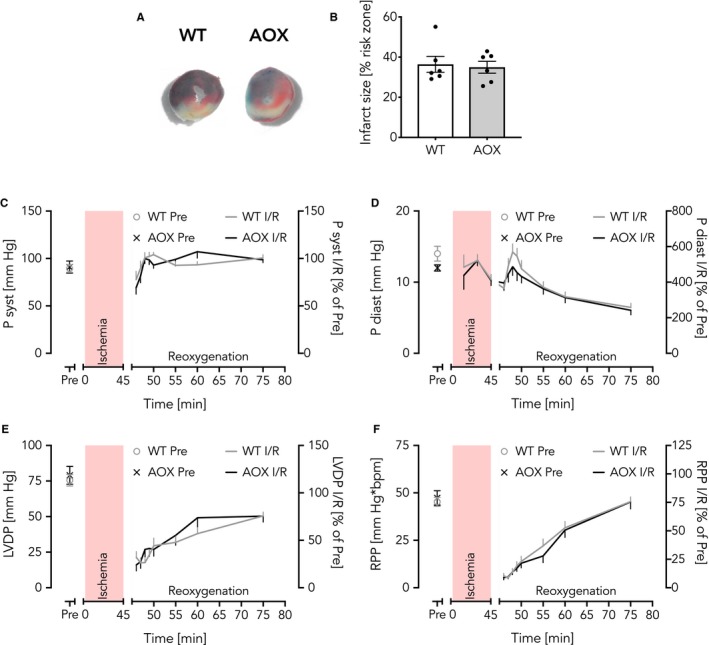
AOX does not protect the heart from I/R injury. A, B, Open‐chest in situ mouse heart model with 30 min ischaemia and 2 h reperfusion. A, Representative images of infarcts from WT and AOX mice as indicated. Blue, Evans blue staining indicates perfused tissue; brick‐red, TTC converted to precipitate indicates viable post‐ischaemic tissue; white, indicates scar tissue; brick‐red and white together form the 'region of risk' zone. B, Infarct size depicted as % of region at risk zone. Data shown as mean ± SEM of n = 6 experiments. C‐F, Response curves from the ex vivo isolated perfused Langendorff heart model with 45 min ischaemia (red area) and 30 min reoxygenation. C, Systolic pressure (P syst); D, diastolic pressure (P diast); E, left ventricular developed pressure (LVDP); F, heart rate*pressure product (RPP). Pre, control values at time point 0. All Langendorff data are shown as mean ± SEM of n ≥ 8 experiments

Succinate accumulation during cardiac ischaemia^25^ is one proposed trigger for RET‐mediated ROS production^24^ and supposedly I/R‐mediated injury. AOX was previously demonstrated to blunt RET‐induced ROS production under various conditions.^33^, ^34^, ^36^, ^37^ We therefore first tested whether the extent of acute I/R injury (infarct size) would be decreased in the post‐ischaemic heart of mice with excellent cardiac AOX protein expression and catalytic AOX activity.^33^ Using the open‐chest in situ I/R model,^41^ 30 minutes of ischaemia were applied by occluding the left anterior descending (LAD) coronary artery followed by 2 hours of reperfusion in wild‐type (WT) and AOX littermates. The infarct size was estimated as a percentage scar tissue of the risk zone.^41^, ^52^ As shown in Figure 1A,B, AOX expression did not confer any benefit. We next sought to investigate whether the heart might nevertheless benefit during the early post‐ischaemic phase through improved functional recovery of surviving myocytes and analysed heart contractile functions ex vivo in the isolated, perfused (Langendorff) heart. Upon 45 minutes of ischaemia, WT and AOX hearts showed nearly indistinguishable systolic (P syst, Figure 1C) and diastolic (P diast, Figure 1D) pressure‐response curves. Furthermore, calculated left ventricular developed pressures (LVDP [maximal systolic–diastolic pressure]) (Figure 1E) and heart rate*pressure products (RPP, a surrogate value of cardiac work)^53^ (Figure 1F) revealed no difference. Of note, hearts typically develop a rigour during ischaemia. This manifests as an increase in pressure during the ischaemic insult and, in our experience, correlates closely with the infarct size. WT and AOX hearts developed the same degree of rigour during ischaemia (Figure 1D) once again being indicative for a lack AOX‐mediated cardioprotection during I/R.

### 3.2 AOX is catalytically active in post‐anoxic heart mitochondria

**Figure 2:**
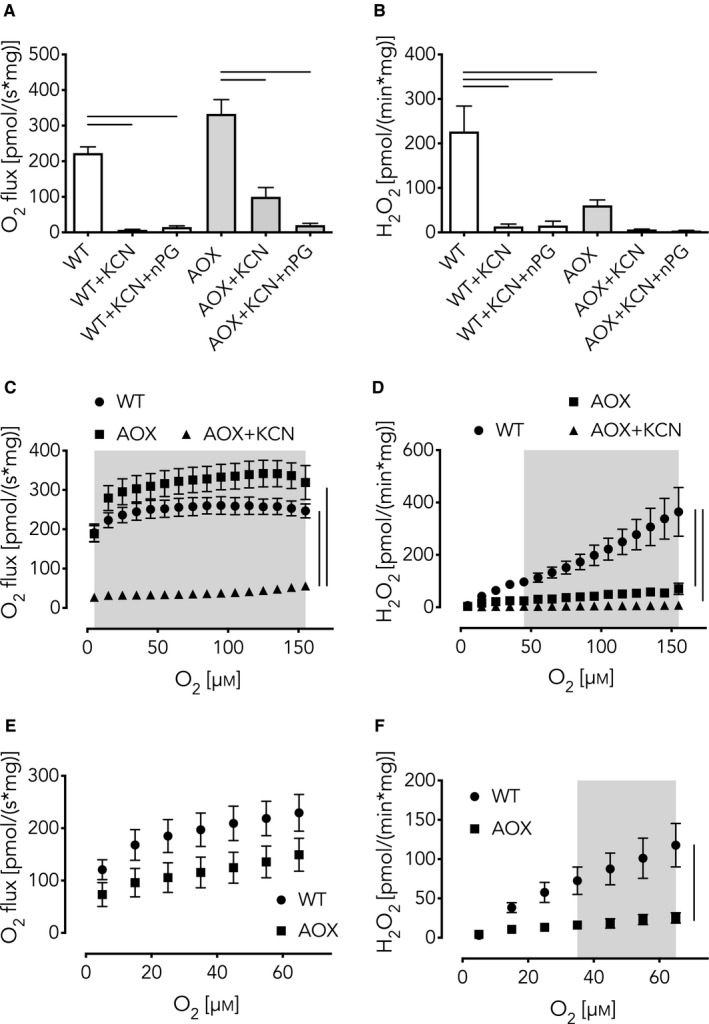
AOX is catalytically engaged in post‐anoxic heart mitochondria and lowers mitochondrial ROS production. A‐F, Isolated WT and AOX heart mitochondria energized with cII substrate succinate and addition of inhibitors as indicated. KCN, cIV inhibitor potassium cyanide; nPG, AOX inhibitor *n*‐propyl gallate. A, Oxygen consumption. B, Hydrogen peroxide production. Data in (A, B) are shown as mean ± SEM of n ≥ 3 experiments. Horizontal bars in (A, B) indicate significant differences with *P* < .05 analysed by 1way ANOVA and Tukey's multiple comparisons test. C, Oxygen consumption in dependence of oxygen concentration. D, Hydrogen peroxide production in dependence of oxygen concentration. E, Oxygen consumption during reoxygenation after 20 min of anoxia. F, Hydrogen peroxide production during reoxygenation after 20 min of anoxia. Data in (C‐F) are shown as mean ± SEM of n ≥ 3 experiments. Grey areas and vertical bars indicate significant differences with *P* < .05 analysed by 2way ANOVA and Tukey's multiple comparisons test in (C, D) and Sidak's multiple comparisons test in (F)

One possible explanation for the observed failure of protection could be insufficient AOX expression or its catalytic impairment in the post‐ischaemic heart. To test this possibility, we isolated mitochondria from WT and AOX mouse hearts and measured oxygen consumption and ROS production in vitro, and, importantly, confirmed a robust expression of AOX by Western blot (Figure S1A). As expected, AOX supported succinate‐driven respiration in a cyanide‐resistant manner (Figure 2A) at ambient oxygen concentrations. AOX‐driven cyanide‐resistant respiration was associated with the prevention of mitochondrial hydrogen peroxide production (Figure 2B). Next, we tested whether AOX catalytic activity depends on a certain oxygen concentration. In other words, we sought to test if AOX loses its catalytic activity underneath a critical threshold of oxygen availability such as seen in the ischaemic heart. We thus measured oxygen consumption and hydrogen peroxide production under different oxygen concentrations ranging from ~21% (ambient air) to full anoxia (Figure 2C,D). We found that the oxygen consumption in AOX heart mitochondria was slightly higher than in WT across a broad range of oxygen concentrations (Figure 2C). Hydrogen peroxide production showed a linear relationship with oxygen availability in WT and was lower in AOX mitochondria (Figure 2D). To simulate the situation in the post‐ischaemic heart, we challenged heart mitochondria by a 20 minutes period of anoxia (simulated ischaemia, Figure 2E,F). Using this approach, oxygen consumption in WT heart mitochondria was slightly higher than in those from AOX hearts, at all oxygen concentrations (Figure 2E). In contrast, hydrogen peroxide production, which again showed a linear relationship with oxygen availability, was significantly lower in AOX heart mitochondria (Figure 2F). To rule out the possibility that the period of anoxia was not sufficient, we replicated the experiments with an extended period of anoxia (30 minutes), which essentially gave the same results (Figure S1B‐E). Together with previous findings of AOX activity in the heart under various conditions,^33^, ^38^, ^40^ we concluded that a functional impairment of AOX is an unlikely explanation for the lack of cardioprotection against I/R injury, and that, therefore, all observed phenomena can mechanistically be attributed to catalytic AOX activity interfering with the mitochondrial respiratory chain and specifically to the restoration of the electron flux through the ETC.

### 3.3 AOX improves mitochondrial functions 3 weeks after an ischaemic insult

**Figure 3:**
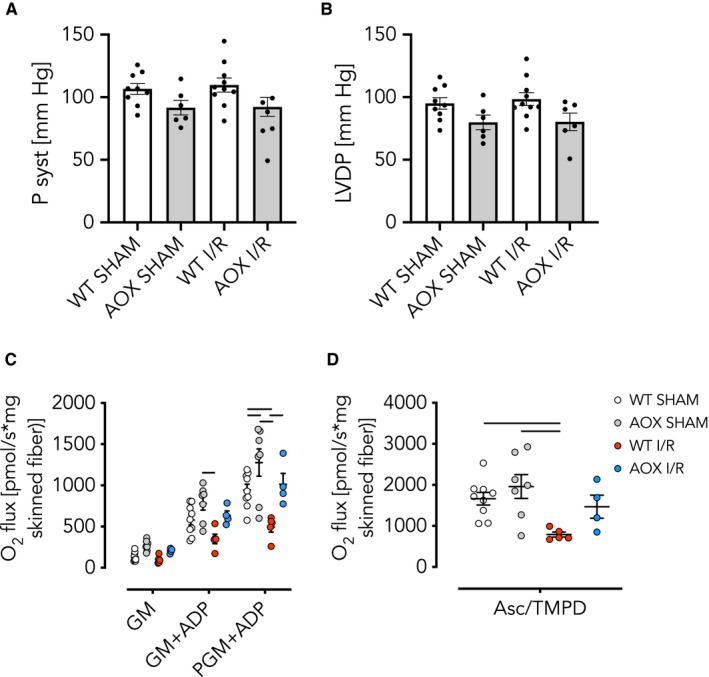
AOX expression does not affect cardiac contractility in vivo 3 wk after I/R despite improved respiratory function. A, B, Left ventricular pressures built up ex vivo in the isolated perfused Langendorff heart model of WT and AOX hearts subjected to 45 min of ischaemia or SHAM operation followed by 3 wk of reperfusion and remodelling in vivo. Data shown as mean ± SEM of n ≥ 6 experiments. A, Systolic pressure (P syst); B, left ventricular developed pressure (LVDP). C, D, Oxygen consumption of isolated skinned heart fibres. Data shown as mean ± SEM of n ≥ 4 experiments. C, Complex I (cI) activity driven by combinations of pyruvate (P), glutamate (G) and malate (M) as indicated, plus ADP. Horizontal bars indicate significant differences with *P* < .05 analysed by 2way ANOVA and Tukey's multiple comparisons test. D, Complex IV (cIV) activity driven by ascorbate (Asc) and *N*,*N*,*N*′,*N*′‐tetramethyl‐*p*‐phenylenediamine (TMPD). Horizontal bars indicate significant differences with *P* < .05 analysed by 1way ANOVA and Tukey's multiple comparisons test

We hypothesized that the restoration of electron flux by AOX and supposed decrease in RET‐induced ROS, despite the lack of a measurable advantage during the acute phase, might yet be beneficial for the post‐ischaemic remodelling process. We tested this assumption in WT and AOX mice 3 weeks after a transient (45 minutes) ischaemic insult followed by restoration of blood flow (reperfusion).^54^ Functional left‐heart parameters (P syst and LVDP) measured ex vivo, however, revealed no significant difference between WT and AOX (Figure 3A,B). Conversely, high‐resolution respirometry using permeabilized heart fibres revealed that cI‐linked respiration, driven by pyruvate, glutamate and malate (PGM) plus ADP (Figure 3C), as well as cIV activity driven by ascorbate/TMPD (Figure 3D) was significantly compromised in WT I/R but not AOX I/R hearts. The finding that cIV was impaired in WT I/R hearts came unexpected, since cIV lies downstream of the quinone pool and the ROS‐producing complexes cI and cIII. Interestingly, electron microscopy revealed no evidence for an ultrastructural correlate of these biochemical differences, for example at the level of mitochondrial cristae density (Figure S2).

**Figure 4:**
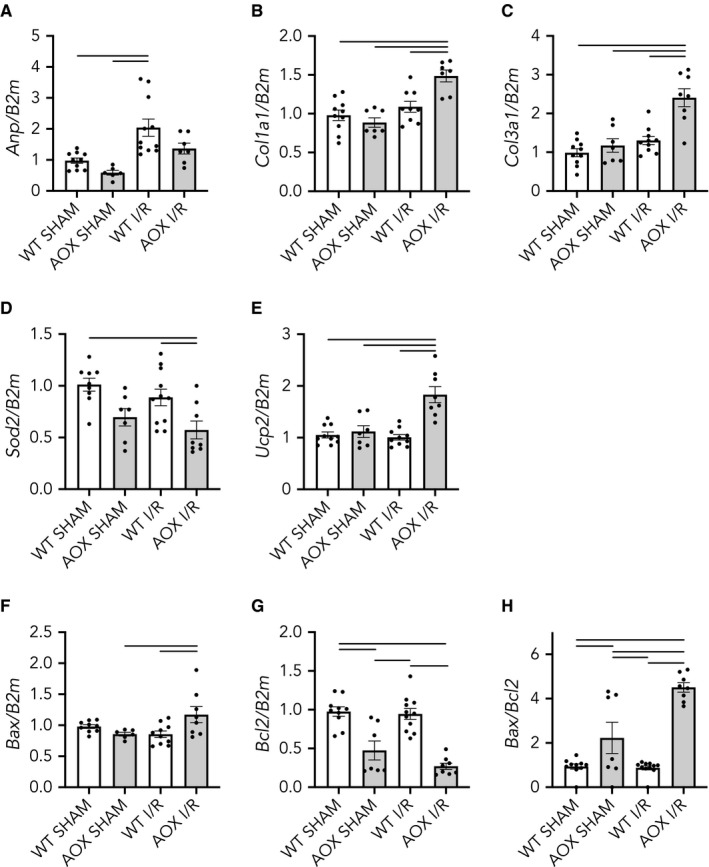
Relative transcript levels in total RNA from WT and AOX hearts subjected to 45 min of ischaemia or SHAM operation followed by 3 wk of reperfusion and remodelling in vivo, normalized to *B2m* (*β_2_ microglobulin*), a reference housekeeping gene. Data shown as mean ± SEM of n ≥ 6 experiments. Horizontal bars indicate significant differences with *P* < .05 analysed by 1way ANOVA and Tukey's multiple comparisons test. A, *Atrial natriuretic peptide* (*Anp*); B, *Collagen 1* (*Col1a1*); C, *Collagen 3* (*Col3a1*); D, *Superoxide dismutase 2* (*Sod2*); E, *Uncoupling protein 2* (*Ucp2*); F, *Bcl2‐associated X* (*Bax*); G, *B‐cell lymphoma 2* (*Bcl2*); H, *Bax*/*Bcl2* expression ratio

Typically, adaptive cardiac response to hemodynamic or metabolic stress is seen as myocardial hypertrophy concomitant with and triggered by an alteration of gene expression. To determine whether respiratory restoration by AOX induced a shift in gene expression in response to the ischaemic insult, we measured transcript levels of common marker genes. First, we measured that of the *atrial natriuretic peptide* (*Anp*), which is recognized as both a biomarker for the diagnosis of heart failure as well as a prognostic marker for cardiovascular risk.^55^ Expression of the *Anp* was significantly increased by I/R in WT hearts but, importantly, this increase was blunted in AOX animals (Figure 4A). Since ANP has been described as profibrogenic^56^ and since excess cardiac collagen synthesis and deposition is known to negatively affect contractile function,^57^ we measured transcripts of *collagen 1* (*Col1a1*) and *3* (*Col3a1*), which we found specifically increased in the AOX I/R group (Figures 4B,C). We also looked at the expression of mitochondrial genes involved in the response to oxidative stress. *Superoxide dismutase 2* (*Sod2*) is a mitochondrial protein that detoxifies superoxide generated as by‐product of OXPHOS, by converting it to hydrogen peroxide and molecular oxygen. *Sod2* is induced as part of the cell‐inherent ROS stress response,^58^ which, when lacking, induces cardiac malfunctioning.^59^ Transcript levels of *Sod2* appeared down‐regulated in AOX hearts compared with WT, irrespective of any intervention (Figure 4D). Uncoupling proteins (UCPs), in contrast, are mitochondrial inner membrane proteins that act as proton leaks, thereby dissipating the proton gradient. *Uncoupling protein 2* (*Ucp2*) has previously been shown to be instrumental for protection against pressure overload‐induced right heart failure.^60^ Here, we found *Ucp2* specifically up‐regulated in the AOX I/R group (Figure 4E). Finally, heart failure due to apoptotic loss of cardiomyocytes has been long discussed.^61^ Bcl‐2‐associated X protein (BAX) is a pro‐apoptotic member of the BCL2 protein family. Indeed, *Bax* transcript expression has been negatively correlated with cardiac function whilst *Bcl2* transcript expression has positive effects.^62^ More specifically, an increased *Bax*/*Bcl2* ratio has been seen in cardiac fibrosis.^63^ Of note, we found a significant increase in the ratio of *Bax* to *Bcl2* (Figure 4F‐H), which is indicative for a cellular shift towards a pro‐apoptotic phenotype.

### 3.4 AOX impairs cardiac contractility 9 weeks after an ischaemic insult

**Figure 5:**
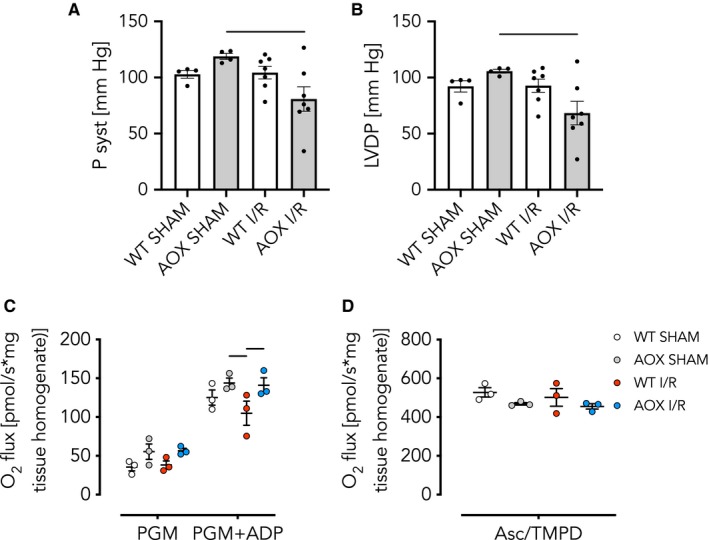
AOX expression decreases cardiac contractility 9 wk after I/R. A, B, Left ventricular pressures built up ex vivo in the isolated perfused Langendorff heart model of WT and AOX hearts subjected to 45 min of ischaemia or SHAM operation and 9 wk of reperfusion/ remodelling. Data shown as mean ± SEM of n ≥ 4 experiments. Horizontal bars in (A) and (B) indicate significant differences with *P* < .05 analysed by 1way ANOVA and Tukey's multiple comparisons test. A, Systolic pressure (P syst); B, left ventricular developed pressure (LVDP). C, D, Oxygen consumption of heart tissue homogenate. Data shown as mean ± SEM of n = 3 experiments. C, Complex I (cI) activity driven by pyruvate (P), glutamate (G) and malate (M) plus ADP. Horizontal bars indicate significant differences with *P* < .05 analysed by 2way ANOVA and Tukey's multiple comparisons test. D, Complex IV (cIV) activity driven by ascorbate (Asc) and *N*,*N*,*N*′,*N*′‐tetramethyl‐*p*‐phenylenediamine (TMPD)

**Figure 6:**
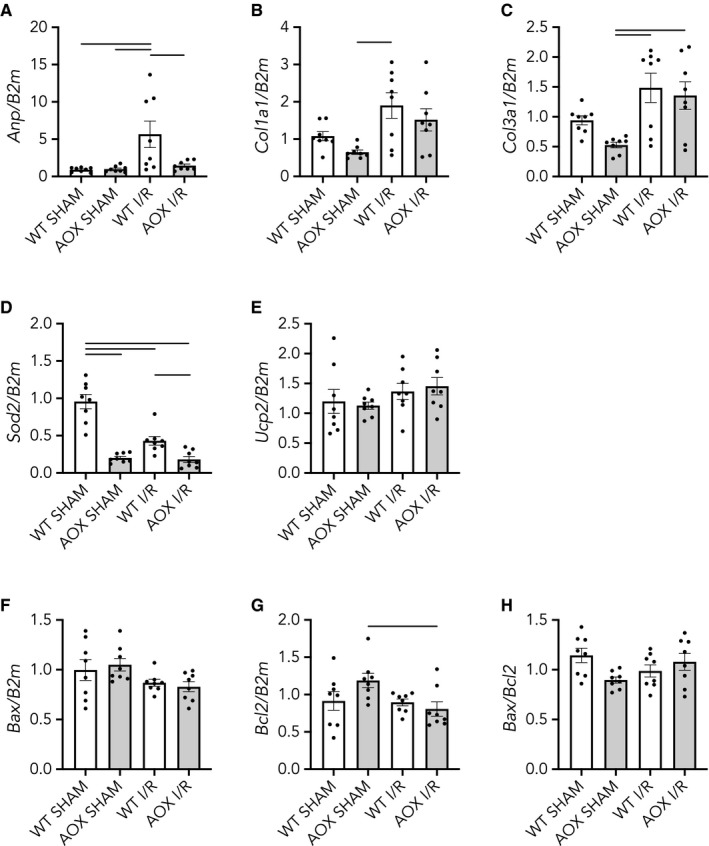
Relative transcript levels in total RNA from WT and AOX hearts subjected to 45 min of ischaemia or SHAM operation followed by 9 wk of reperfusion and remodelling in vivo, normalized to *B2m* (*β_2_ microglobulin*), a reference housekeeping gene. Data shown as mean ± SEM of n = 8 experiments. Horizontal bars indicate significant differences with *P* < .05 analysed by 1way ANOVA and Tukey's multiple comparisons test. A, *Atrial natriuretic peptide* (*Anp*); B, *Collagen 1* (*Col1a1*); C, *Collagen 3* (*Col3a1*); D, *Superoxide dismutase 2* (*Sod2*); E, *Uncoupling protein 2* (*Ucp2*); F, *Bcl2‐associated X* (*Bax*); G, *B‐cell lymphoma 2* (*Bcl2*); H, *Bax*/*Bcl2* expression ratio

The lack of any positive impact on post‐ischaemic functioning, despite the prevention of *Anp* transcript induction, prompted us to investigate the effects of AOX at a later time point. Nine weeks after transient ischaemia (45 minutes), ex vivo functional measurements revealed a significant decrease in P syst (Figure 5A) and LVDP (Figure 5B) specifically in the AOX I/R group. High‐resolution respirometry revealed a continuing, albeit much smaller, cI defect in WT I/R but not in AOX I/R hearts (Figure 5C) whilst cIV activity was fully restored (Figure 5D). At this later time point, *Anp* transcript levels were even more elevated in WT I/R hearts than 3 weeks after ischaemia and AOX again prevented *Anp* induction (Figure 6A). Most other alterations of transcript level were less pronounced (Figure 6B‐H).

### 3.5 AOX fosters extracellular matrix remodelling in the post‐ischaemic heart

To shed light on the mechanisms that may underlie the post‐ischaemic functional deterioration of AOX I/R hearts, we initiated an unbiased, label‐free quantitative proteome approach (data submitted to PRIDE database, identifier PXD014061) and used GOrilla, an online tool designed to discover and visualize enriched gene ontologies in ranked lists^64^, ^65^ (Tables S1 and S2). We revealed a pronounced expression of proteins involved in the reorganization of the extracellular matrix as a major feature in the post‐ischaemic AOX heart. This is best exemplified by the expression of the protein periostin (POSTN, osteoblast‐specific factor). Periostin was up‐regulated almost fivefold in post‐ischaemic compared with SHAM‐operated AOX hearts at 3 weeks, and 17‐fold at 9 weeks, whilst the increase in the corresponding WT hearts was <2‐ and approximately sevenfold after 3 and 9 weeks, respectively.

## 4 DISCUSSION

In the present study, we tested how respiratory restoration by AOX affects the development of I/R injury and adaptive remodelling in the mouse heart. Our data demonstrate that AOX, despite being sufficiently expressed and catalytically active in the healthy and diseased mouse heart,^33^, ^38^, ^40^ fails to confer either acute or chronic cardioprotection, despite respiratory restoration. Instead, AOX expression fosters long‐term adverse remodelling and cardiac contractile impairment. Since one effect of AOX should be a decrease in mitochondrial superoxide production, the outcome of this study may seem surprising. Previous studies using mitochondrially targeted antioxidants under ostensibly comparable conditions did result in cardioprotection, as discussed in detail elsewhere.^66^ Although ischaemic accumulation of succinate was shown to be instrumental for reperfusion injury through RET‐induced ROS production^25^ and despite recent findings that AOX can specifically decrease RET^34^, ^36^, ^37^ the outcome of this study may be in accordance with another proposed mechanism, namely mitohormesis.^13^ For instance, AOX may suppress mitochondrial signals, for example ROS, to a degree that adaptive cardiac remodelling is impaired. Indeed, a cardioprotective effect of low ROS levels has been shown.^14^ Conversely, it may be that AOX, which like cIV requires oxygen as terminal electron acceptor, becomes only slowly reactivated upon tissue reoxygenation. In such a case, cI and/or cIII may produce excess amounts of detrimental ROS in a small post‐ischaemic time‐window before AOX can exert any protective effect. However, our in vitro data indicate that AOX is catalytically engaged both before and after simulated ischaemic insults and prevents RET‐induced ROS production under the conditions experienced in the post‐ischaemic heart.^36^, ^37^ This would argue against a role for slow reactivation. Furthermore, respirometry after 3 weeks of reperfusion revealed that AOX did confer some protection from respiratory impairment, specifically of cI and cIV, despite the lack of any functional improvement. This makes a limitation on AOX catalytic activity in the post‐ischaemic heart an unlikely explanation for the observed lack of acute cardioprotection.

One fundamental difference between the use of antioxidants targeted to mitochondria and genetically expressed AOX is that the former are not normally replenished beyond the acute post‐ischaemic phase. In addition, antioxidants, unlike AOX, may exhibit antioxidant effects during transit to the mitochondria and may therefore not be entirely specific for mitochondrial ROS. An obvious follow‐up study to test this idea would be the use of an inducible AOX strain to enable transient AOX expression during different phases of the I/R procedure. The use of AOX as a therapeutic, in the form of an mRNA mimetic or recombinant protein, may also address this issue. Both approaches would nevertheless suffer other shortcomings such as time‐ and/or organ‐unspecific expression of relevant drivers, unwanted side effects of the same drivers in case of the genetic approach, and immune and inflammatory responses in case of the therapeutic use of AOX, necessitating cumbersome controls.

Despite its ability to maintain respiration and dampen RET‐induced ROS in isolated mitochondria, we found that AOX not only fails to provide cardioprotection but actually promotes post‐ischaemic maladaptation. An interesting aspect of our study is that the presence of AOX in the post‐ischaemic heart restores cI‐driven respiration. In fact, cI may be both, a ROS‐producing complex for instance under conditions as seen in the post‐ischaemic heart^24^, ^25^ or itself a target for ROS damage.^67^ Previously, however, it was shown that supplementing rat hearts undergoing ischaemic insults with Ndi1, a single‐subunit protein that in *Saccharomyces cerevisiae* serves as an NADH dehydrogenase, greatly decreases I/R injury and infarct size.^68^, ^69^ In the light of this and of our findings showing that AOX can restore cI‐driven respiration whilst decreasing ROS, it seems reasonable to conclude that other factors than ROS production or electron flow restoration through cI must contribute to the cardiac stress response.

The present findings have some similarity with another study in which AOX was found to disrupt skeletal muscle remodelling upon knock‐out of cIV assembly factor COX15, a protein involved in the biosynthetic pathway of mitochondrial haem A, a prosthetic group of cIV.^58^ In this model, co‐expression of AOX exacerbated muscle dysfunction and accelerated premature death. The aggravated muscle phenotype was associated with decreased mitochondrial biogenesis and, most likely, impaired progenitor (satellite) cell recruitment. Due to the absence of cardiac progenitor cells, other mechanisms must be considered in the mouse heart, although the underlying mechanism, a failure of regular stress signalling and repair processes, may be similar. Our study also recapitulates a study showing that mitochondria‐targeted overexpression of catalase does not prevent cardioskeletal myopathy in a mouse model of Barth syndrome.^70^ It is important to note, however, that whilst catalase facilitates the turnover of hydrogen peroxide to water and oxygen, AOX prevents the production of superoxide at the impaired ETC and thus acts far upstream.

To provide some indication of the mechanism whereby AOX leads eventually to a maladaptive cardiac remodelling, we studied the expression of a small set of marker genes. Although the findings do not provide a definitive explanation, they reveal valuable clues that should be followed up by a more detailed characterization of the physiological and molecular changes in the AOX I/R model, as now discussed. First, AOX abrogated the up‐regulation of *Anp* at the transcript level. ANP was originally found to be up‐regulated in the failing heart, irrespective of the underlying cause^71^ and was assumed to be compensatory in nature.^72^ This view has subsequently been challenged.^73^ ANP is now considered a diagnostic marker of maladaptive cardiac remodelling.^55^, ^74^, ^75^, ^76^, ^77^ This notion may need further revision, in the light of our finding of increased expression of *Col1a1* and *Col3a1* in AOX hearts, despite the blunted *Anp* response. Whilst collagen decreases wall stress, it also impairs contractile function due to increased stiffness, and accompanies the development of heart failure.^57^

One consequence of *Anp* signalling, shown previously in rat pulmonary arterial smooth muscle cells, is the inhibition of TGF*β*‐induced extracellular matrix components.^78^ We suggest that this might account for the observed up‐regulation of periostin, consistent with another study where heart remodelling upon stress was associated with progressive extracellular matrix remodelling.^79^ However, the role of periostin in cardiac repair remains unclear: whilst it has been found to initiate cell cycle re‐entry of adult cardiomyocytes upon stress^80^ it is also associated with myocardial fibrosis in some forms of heart failure.^81^, ^82^, ^83^ Consistent with the idea that AOX interferes with a respiratory stress signal related to mitochondrial ROS, we observed downregulation of the oxidative stress marker *Sod2,* but up‐regulation of *Ucp2*, previously observed in conditions of cardiovascular stress.^84^

In conclusion, we infer that mitochondrial respiratory restoration and the presumed decrease of mitochondrial ROS by AOX in the post‐ischaemic heart are not sufficient to confer cardioprotection. Instead, AOX expression interferes with adaptive organ remodelling leading to contractile failure, implying an essential role for ETC‐derived stress signals in this process.

## CONFLICT OF INTEREST

MSz has financial interests in developing therapeutics based on *Ciona intestinalis* AOX. All other authors declare no competing interests.

## AUTHOR CONTRIBUTIONS

MSz, HTJ, K‐DS, FNG, TK and MPM conceived and designed experiments and supervised parts of the work. MSz, RS, ZG, ED, MW, PKD, GD‐V, JH, IW, TAN, UG, ARH, VP and CV performed experiments and analysed data. MSz, IW, TK, MPM, TB, FNG, K‐DS and HTJ interpreted data and drafted the manuscript. All authors read and revised the manuscript and contributed substantially to the work.

## Data Availability

All data needed to evaluate the conclusions in the paper are present in the paper and/or the supplements. Additional data related to this paper may be requested from the authors.
