# Supplementary material for: Respiratory chain signalling is essential for adaptive remodelling following cardiac ischaemia
Source: J Cell Mol Med. 2020 Feb 10;24(6):3534–48. doi: 10.1111/jcmm.15043 (PMC7131948; doi:10.1111/jcmm.15043)
Supplement: Supplementary file 3 [file JCMM-24-3534-s003.docx]

**Respiratory chain signaling is essential for adaptive remodeling following cardiac ischemia**

*Szibor et al.*

**Supplementary Information**

Supplementary Figures and Legends

**Supplementary Figure I** (relates to Figure 2)

AOX is catalytically engaged in post-anoxic heart mitochondria and lowers mitochondrial ROS production. (**A**) Western blot of isolated heart mitochondria immunostained for alternative oxidase (AOX) and voltage-dependent anion channel (VDAC1) serving as a loading control. (**B-E**) Isolated WT and AOX heart mitochondria energized with cII substrate succinate. (**B**) Oxygen consumption in dependence of oxygen concentration. (**C**) Hydrogen peroxide production in dependence of oxygen concentration. (**D**) Oxygen consumption during reoxygenation after 30 min of anoxia. (**E**) Hydrogen peroxide production during reoxygenation after 30 min of anoxia. Data in (**B-E**) are shown as mean ± SEM of n = 3 experiments. Gray areas and vertical bars indicate significant differences with *P* < 0.05 analyzed by 2way ANOVA and Sidak's multiple comparisons test.

**Supplementary Figure II** (relates to Figure 3 and Figure 5)

Transmission electron microscopy on mitochondria from heart apex tissue. WT and AOX as indicated. I/R, 45 min of ischemia and 3 weeks or 9 weeks of reperfusion/adaptive remodeling as indicated; SHAM operation served as control. Rare ultrastructural abnormalities best described as paracrystalline inclusions, onion-shaped concentric layering of cristae membranes and matrix compartmentalization equally present in all groups (inlets).
